# Rasch analysis of the long-term conditions questionnaire (LTCQ) and development of a short-form (LTCQ-8)

**DOI:** 10.1186/s12955-020-01626-3

**Published:** 2020-11-30

**Authors:** Laurie Batchelder, Diane Fox, Caroline M. Potter, Michele Peters, Karen Jones, Julien E. Forder, Ray Fitzpatrick

**Affiliations:** 1 Personal Social Services Research Unit, School of Social Policy, Sociology, and Social Research, University of Kent, Canterbury, Kent CT2 7NF UK; 2 Health Services Research Unit, The University of Oxford, Richard Doll Building, Old Road Campus, Roosevelt Drive, Headington, Oxford, OX3 7LF UK; 3 NIHR Collaboration for Leadership in Applied Health Research and Care (CLAHRC) Oxford, Oxford, UK

**Keywords:** Quality of life, Patient-reported outcome measures, Health and social care, Rasch analysis, Long-term conditions

## Abstract

**Background:**

The aim of the current study was to evaluate the structural validity of the 20-item long-term conditions questionnaire (LTCQ) and to explore a potential short-form version of the scale using Rasch analysis.

**Methods:**

Data were collected through postal surveys (February 2016–January 2017) from a sample of 1,211 participants diagnosed with at least one long-term condition (LTC). Identified participants were invited through either local authorities for a social care cohort (n = 294) or primary care practices for a health care cohort (n = 917). Participants were mailed a survey, including the LTCQ, demographic questions, a comorbidities measure, and other validated outcome measures. Respondents were invited to complete a follow-up survey including the LTCQ for assessment of reproducibility.

**Results:**

The main assumptions of the Rasch model from the LTCQ were fulfilled, although infit and outfit indices indicated some items showed misfit. Misfitted items, items that did not have a preceding set or showed some local dependence were removed one at a time, with the remaining candidate items to form an 8-item short version, the LTCQ-8. The Rasch model for the LTCQ-8 explained 64% variance and had a reliability estimate greater than 0.80. Several items in the LTCQ showed uniform differential item function (DIF) in relation to the number of reported LTCs, age, cohort and type of LTCs, but fewer items exhibited DIF in the LTCQ-8. Spearman’s rho correlations between the LTCQ and the LTCQ-8 were strong across the total sample and various subgroups. Correlations between the LTCQ-8 and all reference measures were moderate to strong, and comparable to correlations found between the LTCQ and these measures.

**Conclusions:**

The LTCQ measures a unidimensional construct, and it is therefore acceptable to use a summed total score. The LTCQ-8 also met the assumption of unidimensionality and had comparable construct validity with the LTCQ. Additional validation is required in an independent sample.

## Introduction

Over 15 million people in England (30% of the population) report having at least one long-term condition (LTC) and are major users of health and social care services [1]. Given the prevalence and costs of LTCs, it is a priority of the English government and other countries, including the US and Sweden, to focus on patient-centred outcomes in patients with LTCs [2–4]. To assess and monitor outcomes in individuals with LTCs, measures, such as patient-reported outcome measures (PROMs), are needed [4–6]. PROMs have been developed with the intention to narrow the gap between clinicians’ and patients’ views of the impact of illness. PROMs are routinely used in clinical trials and intervention studies to evaluate patients’ health status and health-related quality of life (HRQoL) [7].

There are two types of PROMs widely indexed within the literature: disease-specific and generic PROMs. The former focus on assessing health outcomes of a specific condition, whereas generic PROMs assess general aspects of functioning and/or well-being and can be used across different patient populations. Although disease-specific PROMs capture more detailed aspects of specific illnesses and tend to be more sensitive to change, the scores are not comparable across different LTC populations, nor do they take account of multi-morbidity [8]. Existing generic PROMs, such as the SF-36 or EQ-5D, are applicable across different populations; however they do not fully capture outcomes that are important in LTCs and may not be appropriate for long-term monitoring of LTCs, particularly for conditions where aspects of HRQoL, such as functional status, might be expected to decline over time [9, 10].

The Long-Term Conditions Questionnaire (LTCQ) was developed to address these limitations [11–14]. The LTCQ is a 20-item PROM developed to assess and monitor outcomes in patients with either single or multiple LTCs (physical and/or mental health condition(s)) and is applicable in both health and social care contexts. The LTCQ was developed through in-depth qualitative interviews with professional and lay stakeholders [11], qualitative interviews with patients with LTCs [13], and cognitive testing with patients with LTCs and professional and lay stakeholders to further refine the items [12]. The LTCQ includes items capturing the broad experience of ‘living well with a LTC(s)’ measured across three broad concepts: impact of LTCs, experience of services and support, and self-care [13]. Items are scored on a scale from 0 (most negative response) to 4 (most positive response). Items 9–15 are negatively phrased and reverse-scored. The LTCQ total score is calculated by summing the item scores and recalibrating the sum to give an overall LTCQ score, ranging from 0–100. Higher scores indicate a better level of ‘living well’ [14].

The LTCQ was validated using factor analysis (principal axis factoring (PAF) and parallel analysis), and results produced a one-factor solution, providing initial evidence of unidimensionality and supporting a total summed score capturing the broad construct of ‘living well with LTC(s)’. Further evidence showed excellent test re-test reliability and construct validity, showing the LTCQ to be a reliable and valid PROM for use in both health and social care [14] (please see [14] for full initial psychometric assessment of the LTCQ, including frequencies of LTCQ items).

Although recent results are promising and already have implications for policy and practice, additional structural validity work of the LTCQ is needed. The work to-date focuses on the application of classical test theory (CTT) [15]. Although CTT is an established psychometric approach that has been used and cited widely within the PROMs literature [16–18], ordinal responses tend to be treated as interval data, where all items are seen as equally “difficult” (severe) [19]. Most outcome measures tend to be ordinal in nature, and their items may vary in terms of their severity or impact, so further evaluation of the LTCQ using robust item-level techniques along with CTT methods is desirable [16]. In comparison to CTT, item response theory (IRT) and Rasch analysis provide more detailed measurement and diagnostic information to identify problematic items and to help improve the scale’s performance [20]. For instance, evidence suggests that when comparing CTT, IRT and Rasch analysis on a PROM (VFQ-25) in a single dataset, all three methods showed similar levels of validity; however IRT and Rasch analysis provided additional measurement details (i.e. rating scale function, misfit, local dependence) [20].

Potter et al. [14] identified some items exhibiting lower internal consistency (i.e. item 16—knowledge, corrected item-total correlation: *r* = 0.35) compared to other items (i.e. item 4—control, corrected item-total correlation: *r* = 0.83). It is possible that items with lower internal consistency could be measuring different constructs, whereas items with high internal consistency could suggest a degree of redundancy. Such items could potentially be removed and allow for creation of a short-form with the remaining items. Clinicians and local authorities work in time-restricted environments, so the availability of a short-form of this measure would be advantageous in certain situations to allow for efficient measurement of outcomes. Although the LTCQ is not a very long PROM, the original measure might be best used at the level of individual decision-making and care planning, whereas a short-form might be preferred for large-scale monitoring or used for special patient populations. Reducing the LTCQ to a short-form may also be beneficial because it would aim to include key items without losing coverage of the overall construct of the scale when administered either on its own or as part of a multipurpose battery of different measures [21]. A shorter measure could also reduce the burden of response, particularly for older participants or those with higher needs.

To further strengthen the LTCQ as a holistic measure of ‘living well’ in both health and social care, we took a pragmatic approach at further examining the psychometric properties of the LTCQ and potentially reducing the length of the measure by using Rasch analysis as a method to better understand the performance of each item.

## Methods

### Participant sample

Previously described by Potter et al. [14], data were collected through two postal surveys (a main survey (Survey 1) and a follow-up survey (Survey 2)) and through two cohorts (a health care cohort and a social care cohort). For the health care cohort, fifteen primary care practices/general practitioners (GPs) from both rural and urban areas invited potential participants from three regions of England (South East, North West, Yorkshire & Humber). Primary care practices invited eligible individuals with a confirmed diagnosis of at least one of eleven specified LTCs (physical and/or mental health conditions) to take part in the study. In a previous study, a list of LTCs were selected from the Quality and Outcomes Framework [22] and other key compendiums of LTCs by a panel of stakeholders [13] (see Potter et al. [14] for further details). Individuals diagnosed more than 12 months prior to beginning the study, those 18 years of age and above, individuals able to provide consent, and those able to communicate in English were eligible. Between February and July 2016, 2,983 eligible participants were invited to take part in the study for the health care cohort.

For the social care cohort, four local authorities (LAs) of different types (unitary, metropolitan, county and London borough) that provide funding for social care services invited potential participants from four geographically diverse regions (North West, East of England, South West, Greater London) (see Potter et al. [14] for further details). Potential participants were eligible if they received community-based services based on the Short and Long Term (SALT) mandatory data returns for social care [23], were 18 years of age and above, were able to provide consent, and were able to communicate in English. Between July 2016 and January 2017, 2,294 eligible participants were invited to take part in the study for the social care cohort.

### Measures

The study packs contained an invitation letter from the GP/LA, the participant information sheet, Survey 1 and an address slip where participants were able to express willingness to take part in the follow-up survey. As outlined by Potter et al. [14], Survey 1 contained the LTCQ, alongside several validated measures to test the LTCQ’s construct validity. These measures included: the EQ-5D-5L including the EQ-VAS [24], Self-efficacy for Managing Chronic Disease 6-item scale [25], an Activities of Daily Living scale [26], and burden of morbidity scale (adapted with permission from the developers to include all conditions for participants recruited) [27]. The social care cohort also completed a measure of social care-related quality of life, the Adult Social Care Outcomes Toolkit (ASCOT) [28]. Supplementary questions included demographics, service use, whether help was needed to complete the questionnaire and an open-response text box for comments.

### Procedure

The research team provided the GPs/LAs with packs containing all study materials, which were mailed directly by the GPs/LAs to the eligible participants, to ensure that no personal data of individuals were disclosed to the research team.

### Statistical analysis

#### Rasch analysis

The Rasch method was applied to the LTCQ as a means to further examine the LTCQ’s psychometric properties and to identify candidate items to form a short-form. The Rasch rating scale analysis [29] model was estimated using WINSTEPS software version 3.92.0 [30]. Rasch analysis [31] is a strict psychometric technique with its assumptions and functional form used to assess whether a single latent trait drives item responses in a questionnaire. The Rasch model shows the assumed probability of participants’ response patterns to items on the scale, which are summed together and tested against a probabilistic model [19, 31]. The Rasch rating scale analysis model is used when a set of items share a fixed set of response rating scale format (e.g. Likert scale), and the relative difficulties of the thresholds do not vary across items. The Rasch rating scale model assumes that the probability of a participant affirming an item is a logistic function of the relative distance between the item location parameter (“item difficulty” or “severity”) and the participant location parameter (“person ability”). The function of that difference is modelled and transformed into a latent trait on a linear scale using log odds [31]. Person ability and item severity are parameters on the logit scale that maximise the likelihood of the observed data, given the functional form specified by the Rasch rating scale model. Item severity and the relative severity of the thresholds indicate the difficulty of endorsing each response category for each item, with the thresholds not varying across items. A formula for the rating scale model is outlined below:

$${{P_{ih} (\theta ) = e^{{\omega_{i} \theta - a_{i} h}} } \mathord{\left/ {\vphantom {{P_{ih} (\theta ) = e^{{\omega_{i} \theta - a_{i} h}} } {\sum\nolimits_{h = 1}^{m} {e^{{\omega_{i} \theta - a_{i} h}} } }}} \right. \kern-\nulldelimiterspace} {\sum\nolimits_{h = 1}^{m} {e^{{\omega_{i} \theta - a_{i} h}} } }}$$

Responses in the rating scale function depend on each individual for the value of the ability parameter $\theta$, describing the individual. In the formula, the response functions specify that *w*_*i*_ refer to the response category scores, which prescribe how the *m* response categories in the measure are scored, while *a*_*ih*_ are the item parameters connected to the items and categories.

Items along the logit scale are ordered based on level of difficulty or severity of items, where items at the top of the logit scale indicate greater item difficulty or severity. Rasch [31] argues that if the person’s ability is higher than the item’s severity, the person is more likely to affirm this item. Conversely, if the person’s ability is lower than the item’s severity, it is predicted that the person is less likely to endorse the most positive response option. For this study, the logarithmic transformation results in an estimation between item location parameter (“item difficulty” or “severity”) and each person’s level of living well with LTC(s) (“person ability”), along a linear scale. The Rasch model further presumes that responses in the questionnaire assess a single construct (i.e. assume unidimensionality). All items in the LTCQ were presumed to assess the experience of ‘living well with LTC(s)’ based on a previous parallel analysis reported by Potter et al. [14].

Seven participants (0.6%) did not complete any of the LTCQ items, and were excluded from the analysis. Participants were included in the Rasch analysis if they answered at least one LTCQ item (1204 participants). CTT approaches involve removing participants who did not answer a certain percentage of questions in the scale. Rasch analysis models responses at the individual response-level. No assumptions are made about the parameter distributions or the absences of individual responses. The model acts if the option was never presented to the individual participant, and missing responses are ignored. This method is especially useful for analysing responses through computerized-adaptive testing [32]. Evidence shows that when comparing this approach to other imputation methods used to handle missing data on a measure of HRQoL, parameter estimations were similar [33].

Table 1 outlines item fit statistics and criteria considered for the current study. An iterative approach was used to examine the psychometric properties of the LTCQ and to identify candidate items for creating a short-form. Rating scale functioning was first examined. All category measures on each item were expected to advance monotonically (i.e. have a preceding set), have at least 10 observations per category [34, page 527], and have an outfit mean square value (MNSQ) for each item response category < 2.0 [35]. Outfit indicates outlier-sensitive fit, whereas infit refers to information-weighted fit [34]. Misfitted items (infit or outfit) were removed one at a time, with the worst-fitted item removed first before repeating the analysis.

**Table 1:** Overview of the Rasch analytic process

Steps	Psychometric property	Aim	Criterion
1	Rating scale function	Assess the scale’s functionality, i.e. do the category measures on each item advance monotonically	Goodness-of-fit: < 2.0 outfit MNSQ, minimum 10 participants per value per item
2	Internal scale validity	Examine how well the item responses match the expected responses in the Rasch model	Item goodness-of-fit: ≤ 1.2 MNSQ, worst fitting item removed one at a time and model subsequently re-run
3	Dimensionality	Assess if the scale measures a single construct	> 50% total variance explained by 1^st^ component (Rasch model), additional components ≤ 5% (or eigenvalue ≤ 2.0) after removal of first component. No more than 1 out of 20 (or 5%) of the residual correlations > 0.30
4	Reliability	Person-separation validity: Assess if the scale can discriminate participants’ responses into groups based on performance; Internal consistency: Assess if the item responses are consistent	Person-separation index: ≥ 2.0 Internal consistency: Cronbach’s alpha ≥ 0.80
5	Differential item functioning (DIF)	Examine how the scale functions among various groups (number of LTCs, age, gender, cohort, hospital admissions)	DIF contrast < 0.43 logits: *p* > 0.01

Item severity was expected to range between − 3.00 to 3.00 logits [19]. Item infit and outfit MNSQ values ≤ 1.2 were considered adequate, and any items showing violations of fit (> 1.2 indicating inconsistent responses) were deleted, with items showing the greatest level of misfit deleted first [36].

Unidimensionality was also assessed through principal component analysis (PCA) of residuals, with a criteria of at least 50% of the variance explaining the Rasch model, with any additional component explaining < 5% (or an eigenvalue ≤ 2.0) of the remaining variance of residuals after removing the first component [34].

Person-separation reliability discriminates participants’ responses into groups based on their performance on the scale. A cut-off criterion of ≥ 2.00 was used, indicating that the scale can distinguish greater than three groups of individuals with different levels of ‘living well with LTC(s)’ [37]. A Cronbach’s alpha cut-off criterion ≥ 0.80 was also considered adequate reliability.

Uniform differential item functioning (DIF) analysis was used to assess item calibrations across key demographic variables: age, gender, the total number of reported LTCs, the number of physical LTCs, the number of mental LTCs, cohort type, and the number of hospital admissions due to a LTC(s). Magnitude of DIF was examined using Zwick, Thayer & Lewis’ Bayes approach to the Mantel–Haenszel statistic [32]. A DIF contrast significant cut-off criterion (slight to moderate significance) of ≥ 0.43 logits was used, with a Bonferroni correction of *p* = 0.01 [34].

Assessments between the short-form of the LTCQ and the original LTCQ were examined in terms of scale and sub-group comparisons and convergent construct validity using SPSS (version 24) software (SPSS, Inc.). It was expected that the short-form of the LTCQ would have similar scores and levels of correlations to the LTCQ.

## Results

### Sample characteristics

A total of 1211 participants were recruited for the current study (23% response rate). 917 participants were recruited through the primary care cohort (31% response rate), and 294 participants were recruited through the social care cohort (13% response rate). Socio-demographic characteristics are presented in Table 2. The age range was 18 to 102 years, with a mean age of 67 (SD 15.3 years). Fifty-four percent (n = 656) were female, and the sample was mainly white British (n = 1,097, 91%). The majority of the sample also reported a high degree of multi-morbidity, with 1,124 participants (93%) having two or more conditions, and the sample mean being 6.2 LTCs (SD 3.8 LTCs).

**Table 2:** Demographic characteristics of the sample (N = 1211)

Variables	%	N
*Cohort*		
Health care (via primary care)	76	917
Social care (via Local Authority)	24	294
*Age (quartiles)*		
18–59 years	26	313
60 – 69 years	24	291
70 – 79 years	24	293
80 + years	21	260
(Missing)	5	54
*Gender*		
Male	44	528
Female	54	656
(Missing)	2	27
*Ethnicity*		
White British	91	1097
Other White (e.g. Irish, European)	3	38
Black/Black British (e.g. African, Caribbean)	2	18
Asian/Asian British (e.g. Indian, Pakistani)	1	17
Mixed	0.60	8
(Missing)	3	33
*Number of long-term conditions*		
1	5	62
2–4	28	344
5 or more	65	780
(Missing)	2	25
*Number of physical health conditions*		
1	6	77
2–4	31	370
5 or more	60	723
(Missing or no physical health condition)	3	41
*Number of mental health conditions*		
1	36	435
2 or more	7	87
(Missing or no mental health condition)	57	689

### Rasch analysis

Rating scale functioning of items in the LTCQ was first assessed, and findings revealed two items that did not advance monotonically for the category measures (items 16 & 17). The response categories 0 ‘Never’, 1 ‘Rarely’ and 2 ‘Sometimes’ were reversed for item 16 (knowledge) and response categories 0 ‘Never’ and 1 ‘Rarely’ were reversed for item 17 (social contact). Several items also showed step calibrations were misfitted to the Rasch model after running iterative Rasch analyses (items 1, 7, 9, 14, 18, 19).

Examination of item infit and outfit statistics revealed that there were no substantial deviations from expectations for the majority of items. Only seven items (items 7, 9, 13, 14, 16, 17, 18) were identified as having beyond the means of 1.04 (infit MNSQ) and 1.03 (outfit MNSQ) in the initial model, revealing that scores on these items tended to be inconsistent with overall response patterns in the questionnaire. Misfitted items (infit or outfit) were removed one at a time, with the worst-fitted item removed first before repeating the analysis.

The PCA of the residuals of the LTCQ revealed that the Rasch model explained between 59% and 64.3% of the variance across the different iterations of the Rasch model, indicating unidimensionality across the various models. The first contrast (second dimension) also explained between 5.8% (eigenvalue = 2.77) and 8% (eigenvalue = 1.79) across the different iterations. Results further revealed standardised residual correlations exceeding a cut-off criterion of > 0.30, indicating some local dependence between items (items 1, 2, 3, 5, 6, 20). In combination with item misfit, these items were subsequently removed from the main analysis. It is worth noting that item 7 (safe at home) loaded highest onto the first contrast when testing these seven items as a separate scale; therefore this question was reintroduced on the LTCQ, as it was thought to be theoretically meaningful to include both safety questions within the questionnaire.

Assessment of item and measure reliability further revealed that the person separation index for the LTCQ ranged from 2.93 to 2.17. The Cronbach’s alpha coefficient ranged from 0.90 to 0.82.

After conducting iterative Rasch analyses, candidate items from the LTCQ were identified to form a short-form. Results are presented in Table 3 (for a full review of the iterative Rasch analyses findings, please see Additional file 1). The short-form of the LTCQ included 8 items (items 4, 7, 8, 10, 11, 12, 15, 19) (to be known as the LTCQ-8). Item severity measures for the LTCQ-8 ranged from − 1.58 to 1.32, and item-fit statistics ranged from 0.83 to 1.21. One item (item 10—dependency) showed very slight misfit (1.21), however researchers felt that this item was highly relevant for participants who rely on health and social care services and for the overall construct of the measure. This item also presented satisfactory functioning on all other assessments of item and person fit. The LTCQ-8 further showed that the Rasch model explained 64.3% of the total variance, with the first contrast explaining 8% (eigenvalue = 1.79) of variance. Item and person reliability results for the LTCQ-8 consisted of a person separation index of 2.17 and a Cronbach’s alpha coefficient of 0.82, providing further evidence of a reliable measure of ‘living well with LTC(s)’.

**Table 3:** Final Rasch model results for the short 8-item version of the LTCQ (LTCQ-8) (N = 1204)

Item #	Item description	Item severity measures	Infit MNSQ	Outfit MNSQ
4	Felt in control of daily life	− 0.01	0.83	0.80
7	Felt safe at home	− 1.58	1.06	0.82
8	Felt safe outside the home	− 0.12	0.93	0.86
10	Felt more dependent on others than you wanted*	1.32	1.21	1.20
11	Felt lonely due to health conditions*	0.23	1.13	1.06
12	Worried about being treated differently*	− 0.20	1.13	1.10
15	Felt that your health conditions made you unhappy*	0.76	0.90	0.95
19	Felt confident in managing health conditions	− 0.40	0.90	0.89

*Questions are reverse-scored, i.e. ‘Never’ is the most positive response option

### Differential item functioning

DIF analyses were undertaken by age, gender, the number of reported mental and physical LTCs, cohort type, and the number of hospital admissions due to a LTC(s) on the LTCQ and the LTCQ-8. Table 4 shows the DIF results. Findings showed that both versions of the LTCQ functioned similarly for gender. There were significant differences on one item in the LTCQ, namely item 18 (support) by the number of reported physical health LTCs. For the number of reported mental LTCs, there were significant differences on items 3 and 16. DIF was no longer present in the LTCQ-8 for the number of reported mental and physical LTCs. Significant differences were found on one item by age (item 12—stigma) in the LTCQ. On the LTCQ-8, there were significant differences on two items by age (item 10–dependency and item 12–stigma). Findings showed significant differences on four items between the health and social care cohorts in the LTCQ (items 2, 9, 10, 18). There were a smaller number of significant differences by cohort in the LTCQ-8, with only two items statistically significant (item 10 and 15). Both versions functioned on the number of hospital admissions due to a LTC(s).

**Table 4:** Differential item functioning results for the LTCQ and the LTCQ-8 (N = 1204)

Differential item functioning	LTCQ	LTCQ-8
Gender	No DIF	No DIF
Age (quartiles)	Item 12: more severe for individuals 18–59 (0.46, *p* = 0.001)	Item 10: more severe for individuals 80 + (0.46, *p* = 0.001)
	Item 12: less severe for individuals 80 + (0.45, *p* = 0.001)	Item 12: less severe for individuals 80 + (0.48, *p* = 0.001)
Number of physical health LTCs	Item 18: more severe for individuals with 1 physical health LTC (0.51, *p* = 0.001)	No DIF
Number of mental health LTCs	Item 3: less severe for individuals with 2 or more mental health LTCs (0.46, *p* = 0.001)	No DIF
	Item16: less severe for individuals with 2 or more mental health LTCs (0.56, *p* = 0.001)	
Cohort	Item 2: less severe for individuals in primary care (0.61, *p* = 0.001)	Item 10: less severe for individuals in primary care (0.51, *p* = 0.001)
	Item 9: more severe for individuals in primary care (0.59, *p* = 0.001)	Item 15: less severe for individuals in social care (0.48, *p* = 0.001)
	Item 10: less severe for individuals in primary care (0.60, *p* = 0.001)	
	Item 18: more severe for individuals in primary care (0.44, *p* = 0.001)	
Admitted to hospital in last 12 months due to LTC	No DIF	No DIF

Similar to the LTCQ, a sum of LTCQ-8 was calculated and recalibrated to give an overall score ranging from 0–100, with higher scores indicating a better level of ‘living well with LTC(s)’. Items 10, 11, 12 and 15 are negatively phrased and reverse-scored.

### Scale and sub-group comparisons

The means, standard deviations and distributions (via percentiles) for scores for both versions of the LTCQ are presented in Tables 5 and 6. Findings showed comparable distributions across both versions of the LTCQ in the total sample. These distributions were also comparable across age quartiles, cohort type, whether respondents were admitted to the hospital due to a LTC(s) in the last 12 months, and whether respondents reported mental health conditions (reported/not reported). Findings further showed a strong positive correlation across the total sample. Further correlations within sub-groups across both versions of the LTCQ also revealed strong positive associations between the LTCQ and the LTCQ-8 across different age groups (quartiles), cohort type, whether those who were admitted to the hospital within the last 12 months due to a LTC(s), and whether respondents reported as having either physical or mental health condition(s).

**Table 5:** Comparison of LTCQ scores (completed in full) (LTCQ and LTCQ-8) among sub-groups- cohort, hospital admissions, LTC-type

	Total sample (N = 1211)		Primary care (N = 917)		Social care (N = 294)		LTC hospital admission (N = 230)		No LTC hospital admission (N = 953)		No mental health condition reported (N = 689)		Mental health condition reported (N = 522)	
	LTCQ	LTCQ-8	LTCQ	LTCQ-8	LTCQ	LTCQ-8	LTCQ	LTCQ-8	LTCQ	LTCQ-8	LTCQ	LTCQ-8	LTCQ	LTCQ-8
N	1082	1156	838	894	244	262	197	213	863	917	624	664	458	492
Mean score	65.1	65.0	70.0	70.4	48.2	46.6	54.6	53.2	67.8	68.2	74.2	75.6	52.7	50.8
SD	23.0	25.1	21.7	23.7	19.1	20.7	21.5	23.6	22.7	24.6	20.2	21.1	20.8	23.1
SE	0.7	0.7	0.8	0.8	1.2	1.3	1.5	1.6	0.8	0.8	0.8	0.8	1.0	1.0
25th %	46.3	43.8	53.8	53.1	35	31.3	39.4	37.5	48.8	50.0	60.0	62.5	38.4	34.8
50th %	66.3	65.6	72.5	75.0	46.3	43.8	51.3	50.0	71.3	71.9	78.8	81.3	48.8	46.9
75th %	85.0	87.5	88.5	90.6	58.8	62.5	71.9	71.9	87.5	90.6	91.3	93.8	68.8	68.8
Correlation (Spearman's)	.97***		.96***		.94***		.96***		.97***		.96***		.96***	

***Correlation is significant at *p* < 0.001 (2-tailed)

**Table 6:** Comparison of LTCQ scores (completed in full) (LTCQ and LTCQ-8) among sub-groups- age quartiles

	Age 18–59 (N = 313)		Age 60–69 (N = 291)		Age 70–79 (N = 293)		Age 80 + (N = 260)	
	LTCQ	LTCQ− 8	LTCQ	LTCQ− 8	LTCQ	LTCQ− 8	LTCQ	LTCQ− 8
N	296	307	267	282	265	282	207	233
mean score	57.9	55.5	68.0	68.4	71.5	72.4	63.4	64.4
SD	23.6	26.0	22.2	24.1	22.0	23.1	21.5	23.1
SE	1.4	1.5	1.4	1.4	1.3	1.4	1.5	1.5
25th %	38.8	34.4	48.8	50.0	53.8	53.1	46.3	43.8
50th %	56.3	53.1	71.3	71.9	75.0	75.0	65.0	65.6
75th %	77.2	78.1	86.3	90.6	91.3	93.8	80.0	84.4
Correlation (Spearman's)	.97***		.96***		.97***		.97***	

***Correlation is significant at *p* < 0.001 (2-tailed)

### Convergent construct validity

The sample’s mean scores for the EQ-5D-5L, EQ-VAS, the Lorig self-efficacy scale, the Activities of Daily Living (ADLs) scale, and the Bayliss burden of morbidity scale are presented in Table 7, as well as correlations (Spearman’s rho) between both versions of the LTCQ and all independent measures used to assess the construct of ‘living well with LTC(s)’. Results showed comparable associations between both versions of the LTCQ with independent measures. Findings showed moderate to strong correlations, with positive associations between the LTCQ with scores on the EQ-5D-5L, EQ-VAS and the Lorig self-efficacy scale. Findings also showed negative associations between both versions of the LTCQ and scores on the ADL scale and the Bayliss burden of morbidity scale.

**Table 7:** Convergent construct validity of the LTCQ and the LTCQ-8 with independent measures (Spearman’s rho)

Measure	Mean score (SD, SE, 95% CI)	Score range	Correlation with LTCQ	Correlation with LTCQ-8
LTCQ	65.10 (23.04, 0.70, 63.7–66.5)	0–100	–	0.97***
LTCQ-8	65.01 (25.13, 0.74, 63.6–66.5)	0–100	0.97***	–
EQ-5D-5L	0.62 (0.33, 0.01, 0.60–0.63)	− 0.28–1.00	0.82***	0.80***
EQ-VAS	62.40 (24.65, 0.72, 61.0–63.8)	0–100	0.79***	0.77***
Lorig self-efficacy scale	6.22 (2.71, 0.08, 6.1–6.4)	1–10	0.87***	0.84***
Activities of daily living	4.98 (4.76, 0.14, 4.7–5.3)	0–13	− 0.79***	− 0.77***
Bayliss burden of morbidity	16.44 (13.10, 0.38, 15.7–17.2)	0–150	− 0.64***	− 0.61***

***Correlation is significant at *p* < 0.001 (2-tailed)

## Discussion

The aim of this study was to further examine the structural validity of the LTCQ using Rasch analysis, as a robust test of measuring the construct of ‘living well with LTC(s)’ which led to the identification of a short-form (LTCQ-8). Results revealed that the LTCQ explained 59% variance by the first dimension of ‘living well with LTC(s),’ suggesting that the LTCQ broadly measures a unidimensional construct. Rasch analysis further identified candidate items to be included in a short-form based on how well the items performed using this robust psychometric technique in the validation sample. After assessing items based on item fit and local dependence, a resulting 8-item short-form was formed. The LTCQ-8 exhibited a higher variance proportion (64.3%) explained by the first dimension. The LTCQ-8 further revealed a Cronbach’s alpha coefficient greater than 0.80. Both versions of the LTCQ were able to distinguish at least three distinct groups of participants of degrees of ‘living well with LTC(s)’. Both versions of the LTCQ showed significant DIF on some items, although there were fewer differences in the LTCQ-8. Spearman’s rho correlations between both versions were strong across the total sample, as well within sub-groups. Similar patterns of correlations were also revealed between both versions of the LTCQ and independent outcome measures.

The findings suggest that the LTCQ can be considered a broad unidimensional measure of ‘living well with LTC(s)’ in both versions of the scale. This is the first assessment of unidimensionality of the LTCQ using Rasch analysis, and the results further support recent work assessing the initial validation of the LTCQ [14]. In the initial validation study, results revealed a one-factor solution after undertaking PAF, with an eigenvalue of 2.3 explaining 75% of the variance. The current study further confirms the LTCQ reflects a unidimensional construct broadly capturing the experience of ‘living well with LTC(s)’, which is suitable for use in a diverse sample of health and social users with a range of LTCs.

Using Rasch analysis, this approach has further allowed us to identify candidate items for a short-form of the LTCQ (LTCQ-8) based on how well items fit the Rasch model. In total, 12 items were omitted from the measure. The LTCQ-8 functioned well as a short-form, with initial evidence of a unidimensional structure and strong item and person reliability. There was also further evidence of high correlations between scores on the LTCQ and LTCQ-8 and similar associations between the LTCQ-8 and independent measures to that of the LTCQ. This shows that the proposed LTCQ-8 has comparable structural and construct validity and reliability to the LTCQ. The strong correlation of the LTCQ and LTCQ-8 with the Lorig self-efficacy measure may indicate that there is conceptual overlap between the LTCQ measures and self-efficacy. The LTCQ was developed to capture quality of life and well-being rather than self-efficacy; although some emerging themes and items from the qualitative work [13] address issues of self-efficacy (e.g. items on feeling in control of life or confident in self-management). However, the LTCQ, in particular the long version, address additional concepts to self-efficacy but it would be reasonable to hypothesize that people who are more confident in self-management achieved better quality of life living with a LTC. Future research may further investigate the correlation between core concepts of the LTCQ and self-efficacy scores to further investigate discriminant validity.

This overall approach of using a more robust psychometric technique following an initial factor analysis, with the aim to assess the measure’s structural validity and to potentially omit misfitted items, is similar to psychometric approaches used for other outcome measures within the literature [38, 39]. The LTCQ-8 may provide the potential for increased precision of measurement required for larger studies of populations of health and social care users. However, for some clinical purposes and item-by-item analyses, all items can be retained and the original LTCQ may be the preferred measure. In other words, the LTCQ may be more appropriate in clinical settings or in other situations, where capturing a wider number of facets of the experience of ‘living well with LTC(s)’ is more relevant.

DIF was also assessed for both versions of the scale, and findings revealed significant DIF for age, the number of LTCs, and by cohort type for the LTCQ; however there was less DIF in the LTCQ-8. Three items had slight significant DIF for age and cohort type for the LTCQ-8 (items 10—dependency, 12—stigma and 15—unhappiness). Research suggests that significant DIF by sub-group is not uncommon and may indicate a need to develop customized policy strategies for specific sub-groups [40–42]. Further evidence has shown that there may be benefits using Rasch analysis to further discriminate sub-groups [43]. Two further items, items 10 (dependency) and 15 (unhappiness), had significant slight DIF (0.48 and 0.51, respectively) for cohort type (primary care cohort versus social care cohort). These two items were not removed since they were considered theoretically important and presented acceptable levels of functioning in all other scale parameters [44].

The main strength of this study is the use of an item-level psychometric methodology to further explore the structural properties of the LTCQ and to create a short-form. An additional strength is the direct assessment of the psychometric properties of the LTCQ and the LTCQ-8. This study was based on a diverse sample in terms of the number, type and severity of health conditions reported. Further validating the LTCQ in such a diverse sample allows for routine use of the LTCQ in monitoring outcomes of integrated person-centred care. A further strength is the inclusion of both primary care and social care users showing the LTCQ is suitable for use in both health and social care. The current research also included a number of well-validated independent reference measures to further examine the construct of ‘living well with LTC(s)’ in the LTCQ and the LTCQ-8.

There are some limitations worth noting within the current study. The response rate for the social care cohort was 13%, which was lower compared to the 31% response rate for the primary care cohort, indicating that the social care cohort is not as well-represented compared to the health care cohort [14]. However the response rate for the social care cohort was similar to other studies where the social care sample was the main focus [45]. This study has also identified candidate items for a short-form (LTCQ-8) from the original LTCQ using Rasch analysis, and initial construct validity assessment of potential items forming the LTCQ-8 was also undertaken in the same patient sample. This was done due to time constraints, financial limitations and difficulties in recruiting a large sample size of individuals with LTC(s), particularly for those who receive social care. To date, this is the largest dataset recruited containing LTCQ data, and the analysis needed to be conducted in a sample that represented the diversity of individuals with LTC(s), including both health and social care users. However, additional validity and reliability assessment of the LTCQ-8 cannot be obtained using the same sample. We recommend that the identified items in the LTCQ-8 are separately tested as a proposed short-form in an independent sample. Future work should also include confirming the validity of the LTCQ in an independent sample. The majority of participants in the current sample were also white British, which may limit the generalizability of these findings to the wider population. Future research should include cross-validating the LTCQ with other ethnic groups and in other languages. Lastly, although the sample size in the current study can generate accurate item calibrations, current DIF findings are more speculative given the smaller sub-groups [38].

A number of implications for health and social care policy and practice can be drawn from this work. This is the first time Rasch analysis has been used to assess the psychometric properties of the LTCQ, a PROM designed to capture the impact of ‘living well with LTC(s)’ with those who have long-term physical and/or mental health condition(s) and across health and social care. In line with current policy and practice, both versions of the LTCQ include a number of health- and social care-related items to allow for an integrated evaluation of services. Rasch analysis has also enabled us to identify candidate items for a short-form (LTCQ-8). The LTCQ-8 may further allow for the concise measurement of the construct of ‘living well with LTC(s)’, which may be preferred in contexts where time or other resources are limited. A short-form may be a less burdensome method for capturing the experience of ‘living well with LTC(s)’, particularly for those who are frail or who have complex needs. However future work should include separate testing on the 8 short-form candidate items in an independent sample.

## Conclusion

The Long-Term Conditions Questionnaire (LTCQ) is a new PROM designed to capture the concept of ‘living well’ in patients with physical and/or mental health condition(s) for use across health and social care. Using Rasch analysis, the original LTCQ met the assumption of measuring a unidimensional construct. The robust psychometric assessment identified 8 candidate items to form a short-form, and the resulting LTCQ-8 showed improved psychometric properties. Both versions of the LTCQ showed similar patterns in construct validity. In cases where it is relevant to examine the impact of ‘living well with LTC(s)’ item-by-item, for example to inform clinical practice, it may be more appropriate to use the LTCQ. Conversely the identified items of the LTCQ-8 may be more appropriate for in larger-scale monitoring (which favours shorter measures) or with patient populations for whom completion of PROMs is more difficult. Further assessment of identified items from the LTCQ in an independent sample would yield additional evidence for this measure.

## Supplementary information

**Additional file 1: Table 1**. Results from the iterative Rasch analyses from the current study in 1211 participants. **Table 2**. Fit Indices for Each Item in 1211 participants.

## Data Availability

Only members of the research team (i.e. study authors) have access to the study data. The full anonymised data set was shared between all team members (University of Oxford and University of Kent). Direct access will be granted to authorised representatives from the sponsor or host institution for monitoring and/or audit of the study to ensure compliance with regulations.

## Abbreviations

ADLs: Activities of daily living
ASCOT: Adult Social Care Outcomes Toolkit
CTT: Classical test theory
DIF: Differential item functioning
GP: General practitioner
HRQoL: Health-related quality of life
IRT: Item-response theory
LA: Local authority
LTC: Long-term condition
LTCQ: Long-Term Condition Questionnaire
LTCQ-8: Long-Term Condition Questionnaire—Short-form (8)
MNSQ: Mean-square value
PAF: Principal axis factoring
PCA: Principal component analysis
PROMs: Patient-reported outcome measures
SALT: Short and Long Term
SD: Standard deviation

## References

[1] Long Term Conditions Compendium of Information. Department of Health website. https://www.gov.uk/government/uploads/system/uploads/attachment_data/file/216528/dh_134486.pdf. . Published 2012. Accessed27 Mar 2018.

[2] The Adult Social Care Framework 2014/15. Department of Health:HEALTH; London. 2013. Accessed 27 Mar 2018

[3] The NHS Outcomes Framework 2014/15. Department of Health: HEALTH; London. 2013. Accessed 27 Mar 2018

[4] Nelson EC, Eftimovska E, Lind C, Hager A, Wasson JH, Lindblad S. Patient reported outcome measures in practice. BMJ. 2015;350:g7818–g7818. http://www.bmj.com/cgi/doi/10.1136/bmj.g7818 10.1136/bmj.g7818 25670183

[5] Black N (2013). Patient reported outcome measures could help transform healthcare. BMJ..

[6] Raine R, Fitzpatrick R, Barratt H, Bevan G, Black N, Boaden R, et al. Challenges, solutions and future directions in the evaluation of service innovations in health care and public health. Heal Serv Deliv Res. 2016;4(16):1–136. https://www.journalslibrary.nihr.ac.uk/hsdr/hsdr04160/27227187

[7] Stiggelbout AM, Van der Weijden T, De Wit MPT, Frosch D, Légaré F, Montori VM, et al. Shared decision making: really putting patients at the centre of healthcare. BMJ. .2012;344:e256. http://www.ncbi.nlm.nih.gov/pubmed/22286508 10.1136/bmj.e256 22286508

[8] Mujica-Mota RE, Roberts M, Abel G, Elliott M, Lyratzopoulos G, Roland M, et al. Common patterns of morbidity and multi-morbidity and their impact on health-related quality of life: evidence from a national survey. Qual Life Res. 2015;24(4):909–18. http://link.springer.com/10.1007/s11136-014-0820-7 10.1007/s11136-014-0820-7 PMC4366552 25344816

[9] Matza LS, Boye KS, Stewart KD, Curtis BH, Reaney M, Landrian AS. A qualitative examination of the content validity of the EQ-5D-5L in patients with type 2 diabetes. Health Qual Life Outcomes.. 2015;13(1):192. http://hqlo.biomedcentral.com/articles/10.1186/s12955-015-0373-7 10.1186/s12955-015-0373-7 PMC4665831 26627874

[10] Coulter A. Measuring what matters to patients. BMJ. 2017;356:j816. http://www.ncbi.nlm.nih.gov/pubmed/28219884 10.1136/bmj.j816 28219884

[11] Hunter C, Fitzpatrick R, Jenkinson C, Darlington ASE, Coulter A, Forder JE (2015). Perspectives from health, social care and policy stakeholders on the value of a single self-report outcome measure across long-term conditions: a qualitative study. BMJ Open.

[12] Kelly L, Potter C, Hunter C, Gibbons E, Fitzpatrick R, Jenkinson C, et al. Refinement of the Long-Term Conditions Questionnaire (LTCQ): patient and expert stakeholder opinion. Patient Relat Outcome Meas. 2016;Volume 7:183–93. https://www.dovepress.com/refinement-of-the-long-term-conditions-questionnaire-ltcq-patient-and-peer-reviewed-article-PROM 10.2147/PROM.S116987 PMC5118027 27895523

[13] Peters M, Potter C, Kelly L, Hunter C, Gibbons E, Jenkinson C (2016). The long-term conditions questionnaire: conceptual framework and item development. Patient Relat Outcome Meas.

[14] Potter CM, Batchelder L, A’Court C, Geneen L, Kelly L, Fox D, et al. Long-Term Conditions Questionnaire (LTCQ): initial validation survey among primary care patients and social care recipients in England. BMJ Open. 2017;7(11):e019235. http://www.ncbi.nlm.nih.gov/pubmed/29101153 10.1136/bmjopen-2017-019235 PMC5695378 29101153

[15] Nunnally JC, Bernstein IH. Psychometric theory. McGraw-Hill; 1994. p. 752. https://books.google.co.uk/books/about/Psychometric_theory.html?id=r0fuAAAAMAAJ&redir_esc=y

[16] Tennant A, McKenna SP, Hagell P. Application of Rasch Analysis in the Development and Application of Quality of Life Instruments. Value in Health. 2004 Sep-Oct;7 Suppl 1:S22–6; http://www.valueinhealthjournal.com/article/S1098-3015(10)60232-X/pdf 10.1111/j.1524-4733.2004.7s106.x 15367240

[17] Prieto L, Alonso J, Lamarca R. Classical test theory versus Rasch analysis for quality of life questionnaire reduction. Health Qual Life Outcomes. 2003;1(1):27. http://hqlo.biomedcentral.com/articles/10.1186/1477-7525-1-27 10.1186/1477-7525-1-27 PMC194220 12952544

[18] Cappelleri JC, Jason Lundy J, Hays RD. Overview of classical test theory and item response theory for the quantitative assessment of items in developing patient-reported outcomes measures. Clin Ther. 2014;36(5):648–62. http://www.ncbi.nlm.nih.gov/pubmed/24811753 10.1016/j.clinthera.2014.04.006 PMC4096146 24811753

[19] Boone WJ. Rasch analysis for instrument development: why, when, and how? CBE Life Sci Educ. 2016;15(4). http://www.ncbi.nlm.nih.gov/pubmed/27856555 10.1187/cbe.16-04-0148 PMC5132390 27856555

[20] Petrillo J, Cano SJ, McLeod LD, Coon CD. Using classical test theory, item response theory, and rasch measurement theory to evaluate patient-reported outcome measures: a comparison of worked examples. Value in Health. 2015;18(1):25–34. https://www.sciencedirect.com/science/article/pii/S1098301514047305 10.1016/j.jval.2014.10.005 25595231

[21] Laidlaw K, Kishita N, Shenkin SD, Power MJ (2018). Development of a short form of the attitudes to ageing questionnaire (AAQ). Int J Geriatr Psychiatry..

[22] Quality outcomes framework. NHS Digitital website. 2016. Accessed 27 Mar 2018. http://content.digital.nhs.uk/qof

[23] Short- and Long-Term Support (SALT). NHS digital website. Web Master, United Kingdom; 2016. Accessed 27 Mar 2018. http://content.digital.nhs.uk/datacollections/SALT

[24] Herdman M, Gudex C, Lloyd A, Janssen M, Kind P, Parkin D (2011). Development and preliminary testing of the new five-level version of EQ-5D (EQ-5D-5L). Qual Life Res..

[25] Lorig KR, Sobel DS, Ritter PL, Laurent D, Hobbs M (2001). Effect of a self- management program on patients with chronic disease. Eff Clin Pr..

[26] Blake M, Gray M, Balarajan M, Darton R, Hancock R, Henderson C, et al. Social Care for people age 65+: Questionnaire Documentation Background Introduction to the module. NatCen Social Research. 2010. http://www.natcen.ac.uk/media/205502/social-care-questionnaire.pdf

[27] Bayliss EA, Ellis JL, Steiner JF (2005). Subjective assessments of comorbidity correlate with quality of life health outcomes: Initial validation of a comorbidity assessment instrument. Health Qual Life Outcomes..

[28] Netten A, Burge P, Malley J, Potoglou D, Towers A-M, Brazier J, et al. Outcomes of social care for adults: developing a preference-weighted measure. Health Technol Assess. 2012;16(16). Available from: https://www.journalslibrary.nihr.ac.uk/hta/hta16160/10.3310/hta16160 22459668

[29] Andersen EB (1977). Sufficient statistics and latent trait models. Psychometrika..

[30] Linacre JM. Winsteps Rasch measurement computer program. Beaverton, Oregon; 2015. Available from: winsteps.com.

[31] Rasch G (1960). Studies in mathematical psychology: I. Probabilistic models for some intelligence and attainment tests.

[32] Zwick R, Thayer D, Lewis C (2005). An empirical bayes approach to mantel-haenszel DIF analysis. J Educ Meas..

[33] Linden WJ, Glas CAW (2000). Computerized adaptive testing: theory and practice.

[34] Linacre JM. Winsteps® Rasch measurement computer program User’s Guide. Beaverton, Oregon; 2017. Available from: Winsteps.com

[35] Linacre J (2002). What do Infit and Outfit, mean-square and standardized mean?. Rasch Meas Trans.

[36] Smith RM, Schumacker RE, Bush MJ (1998). Using item mean squares to evaluate fit to the Rasch model. J Outcome Meas..

[37] Fisher WJ (1992). Reliability, separation, strata statistics. Rasch Meas Trans.

[38] Bonsaksen T, Kottorp A, Gay C, Fagermoen M, Lerdal A (2013). Rasch analysis of the general self-efficacy scale in a sample of persons with morbid obesity. Health Qual Life Outcomes..

[39] Hendriks AAJ, Smith SC, Chrysanthaki T, Cano SJ, Black N (2017). DEMQOL and DEMQOL-Proxy: a Rasch analysis. Health Qual Life Outcomes..

[40] Bond TG, Fox CM. Applying the rasch model: fundamental measurement in the human sciences second edition. London: Lawrence Erlbaum Associates; 2007. Available from: https://researchonline.jcu.edu.au/9907/2/9907_Bond_%26_Fox_front_pages.pdf

[41] Henson S, Blandon J, Cranfield J (2010). Difficulty of healthy eating: a Rasch model approach. Soc Sci Med.

[42] Kahler CW, Strong DR, Read JP, Palfai TP, Wood MD (2004). Mapping the continuum of alcohol problems in college students: a Rasch model analysis. Psychol Addict Behav..

[43] Fitzpatrick R, Norquist JM, Jenkinson C, Reeves BC, Morris RW, Murray DW (2004). A comparison of Rasch with Likert scoring to discriminate between patients’ evaluations of total hip replacement surgery. Qual Life Res..

[44] Las Hayas C, Quintana JM, Padierna JA, Bilbao A, Muñoz P (2010). Use of rasch methodology to develop a short version of the Health Related Quality of life for Eating Disorders questionnaire: a prospective study. Health Qual Life Outcomes..

[45] Forder J, Malley J, Rand S, Vadean F, Jones K, Netten A. Identifying the impact of adult social care: Interpreting outcome data for use in the Adult Social Care Outcomes Framework. 2016; Available from: www.qoru.ac.uk

