# Supplementary material for: Rasch analysis of the long-term conditions questionnaire (LTCQ) and development of a short-form (LTCQ-8)
Source: Health Qual Life Outcomes. 2020 Nov 30;18:375. doi: 10.1186/s12955-020-01626-3 (PMC7706038; doi:10.1186/s12955-020-01626-3)
Supplement: Supplementary file 1 — Additional file 1: Table 1. Results from the iterative Rasch analyses from the current study in 1211 participants. Table 2. Fit Indices for Each Item in 1211 participants. [file 12955_2020_1626_MOESM1_ESM.docx]

*Table 1.* Results from the iterative Rasch analyses from the current study in 1211 participants

| **Model** | **Analysis** | **Rating scale function** | **Internal scale validity** | **Dimensionality** | **Reliability and internal consistency** |
| --- | --- | --- | --- | --- | --- |
| 1 | LTCQ – 20 items (full reference of items, see Potter et al. (2017)) | 4 items:  Item 9: >2.0 outfit (last option)  Item 14: >2.0 outfit (first option)  Items 16, 17: >2.0 outfit & items do not advance monotonically | 5 items:  Item 9: Outfit 1.32  Item 11: Outfit 1.21  Item 13: Infit 1.38, Outfit 1.22  Item 14: Infit 1.35, Outfit 1.61  Item 16: Infit 1.96, Outfit 2.70 | 1: 59.0%  2: 5.9%  (eigenvalue=2.9) | Separation index: 2.93  Cronbach’s alpha: 0.9 |
| 2 | 18 items - Removal of items 16 and 17 | 3 items:  Item 9: >2.0 outfit (last option)  Item 14 & 18: >2.0 outfit (first option) | 7 items:  Item 6: Infit 1.28  Item 9: Outfit 1.43  Item 11: Infit 1.29, outfit 1.28  Item 12: Infit 1.21  Item 13: Infit 1.45, outfit 1.29  Item 14: 1.44, outfit 1.76  Item 18: Infit 1.25, outfit 1.50 | 1: 62.1%  2: 5.8%  (eigenvalue=2.77) | Separation index: 2.94  Cronbach’s alpha: 0.9 |
| 3 | Examination of 6 items as a separate scale loading onto the first contrast from Model 2 & showed local dependence:  Items 1, 2, 3, 4, 5, 20 | 1 item:  Item 1: >2.0 outfit (first option) | 1 item:  Item 20: Infit 1.27, outfit 1.25 | 1: 72.6%  2: 7.4%  (eigenvalue=1.62) | Separation index: 2.74  Cronbach’s alpha: 0.88 |
| 4 | Examination of 4 items as a separate scale that loaded highest onto the first contrast from Model 2:  Items 2, 3, 4, 5 | N/A | N/A | 1: 75.1%  2: 10.1%  (eigenvalue=1.62) | Separation index: 2.61  Cronbach’s alpha: 0.87 |
| 5 | 12 items - Remaining scale after removing 6 items as a separate scale and items 16 and 17 | 3 items:  Item 9: >2.0 outfit (last option)  Item 14 & 18: >2.0 outfit (first option) | 2 items:  Item 13: Infit: 1.27  Item 14: 1.25, outfit 1.36 | 1: 60.1%  2: 6.7%  (eigenvalue=2.0) | Separation index: 2.35  Cronbach’s alpha: 0.85 |
| 6 | 13 items – Reintroduced item 4 to remaining scale | 3 items:  Item 9: >2.0 outfit (last option)  Item 14 & 18: >2.0 outfit (first option) | 4 items:  Item 6: Infit 1.21  Item 13: Infit 1.30  Item 14: Infit 1.28, oufit 1.42  Item 18: 1.24 | 1: 60.2%  2: 6.3%  (eigenvalue=2.1) | Separation index: 2.45  Cronbach’s alpha: 0.86 |
| 7 | 12 items – Removal of item 14 | 3 items:  Item 6: >2.0 outfit (first option)  Item 9: >2.0 outfit (last option)  Item 18: >2.0 outfit (first option) | 4 items:  Item 6: Infit 1.24  Item 9: Outfit 1.21  Item 13: Infit 1.39  Item 18: Outfit 1.29 | 1: 61.7%  2: 6.6%  (eigenvalue=2.1) | Separation index: 2.45  Cronbach’s alpha: 0.86 |
| 8 | 11 items – Removal of item 13 | 2 items:  Item 6: >2.0 outfit (first option)  Item 18: >2.0 outfit (first option) | 4 items:  Item 6: Infit 1.25  Item 9: Outfit 1.27  Item 10: Infit 1.21  Item 18: Outfit 1.29 | 1: 63%  2: 6.9%  (eigenvalue=2.1) | Separation index: 2.46  Cronbach’s alpha: 0.86 |
| 9 | 10 items – Removal of item 18 | 3 items:  Item 6: >2.0 outfit (first option)  Item 9: >2.0 outfit (last option)  Item 19: >2.0 outfit (first option) | 2 items:  Item 6: Infit 1.30  Item 9: Outfit 1.29 | 1: 65.5%  2: 7.2%  (eigenvalue=2.0) | Separation index: 2.42  Cronbach’s alpha: 0.85 |
| 10 | 9 items – Removal of item 9 | 2 items:  Item 6: >2.0 outfit (first option)  Item 19: >2.0 outfit (first option) | 2 items:  Item 6: Infit 1.28  Item 10: Infit 1.22, oufit 1.25 | 1: 63.2%  2: 7.8%  (eigenvalue=1.90) | Separation index: 2.17  Cronbach’s alpha: 0.82 |
| 11 | 8 items – removal of item 6 | 1 item:  Item 19: >2.0 outfit (first option) (2.1) | 1 item:  Item 10: Infit 1.21 | 1: 64.3%  2: 8%  (eigenvalue=1.79) | Separation index: 2.17  Cronbach’s alpha: 0.82 |

*Table 2.* Fit Indices for Each Item in 1211 participants

| Model number | Item number | Question | Infit | Outfit |
| --- | --- | --- | --- | --- |

| 1. LTCQ – 20 items (full reference of items, see Potter et al. (2017)) | 1 | LTCQCOPE | 0.60 | 0.62 |
| --- | --- | --- | --- | --- |
|  | 2 | LTCQROLE | 0.77 | 0.73 |
|  | 3 | LTCQPHYS | 0.89 | 0.84 |
|  | 4 | LTCQCONT | 0.66 | 0.60 |
|  | 5 | LTCQENJO | 0.82 | 0.81 |
|  | 6 | LTCQHOME | 1.20 | 1.01 |
|  | 7 | LTCQSAF1 | 0.98 | 0.70 |
|  | 8 | LTCQSAF2 | 0.83 | 0.74 |
|  | 9 | LTCQSYMP | 1.08 | 1.32 |
|  | 10 | LTCQDEPE | 1.17 | 1.13 |
|  | 11 | LTCQLONE | 1.20 | 1.21 |
|  | 12 | LTCQSTIG | 1.14 | 1.08 |
|  | 13 | LTCQSERV | 1.38 | 1.22 |
|  | 14 | LTCQTREA | 1.35 | 1.61 |
|  | 15 | LTCQUNHA | 0.85 | 0.90 |
|  | 16 | LTCQKNOW | 1.96 | 2.70 |
|  | 17 | LTCQSOCI | 1.07 | 1.17 |
|  | 18 | LTCQSUPP | 1.09 | 1.10 |
|  | 19 | LTCQCONF | 0.73 | 0.64 |
|  | 20 | LTCQLIFE | 0.78 | 0.73 |

| 2. 18 items - Removal of items 16 and 17 | 1 | LTCQCOPE | 0.63 | 0.65 |
| --- | --- | --- | --- | --- |
|  | 2 | LTCQROLE | 0.79 | 0.74 |
|  | 3 | LTCQPHYS | 0.92 | 0.88 |
|  | 4 | LTCQCONT | 0.68 | 0.62 |
|  | 5 | LTCQENJO | 0.85 | 0.84 |
|  | 6 | LTCQHOME | 1.28 | 1.09 |
|  | 7 | LTCQSAF1 | 1.04 | 0.77 |
|  | 8 | LTCQSAF2 | 0.87 | 0.78 |
|  | 9 | LTCQSYMP | 1.17 | 1.43 |
|  | 10 | LTCQDEPE | 1.20 | 1.17 |
|  | 11 | LTCQLONE | 1.29 | 1.28 |
|  | 12 | LTCQSTIG | 1.21 | 1.18 |
|  | 13 | LTCQSERV | 1.45 | 1.29 |
|  | 14 | LTCQTREA | 1.44 | 1.76 |
|  | 15 | LTCQUNHA | 0.89 | 0.96 |
|  | 18 | LTCQSUPP | 1.25 | 1.50 |
|  | 19 | LTCQCONF | 0.81 | 0.74 |
|  | 20 | LTCQLIFE | 0.83 | 0.77 |
| 3. Examination of 6 items as a separate scale loading onto the first contrast from Model 2 & showed local dependence:  Items 1, 2, 3, 4, 5, 20 | 1 | LTCQCOPE | 0.98 | 1.13 |
|  | 2 | LTCQROLE | 0.86 | 0.82 |
|  | 3 | LTCQPHYS | 0.95 | 0.91 |
|  | 4 | LTCQCONT | 0.90 | 0.88 |
|  | 5 | LTCQENJO | 0.97 | 1.00 |
|  | 20 | LTCQLIFE | 1.27 | 1.25 |
| 4. Examination of 4 items as a separate scale that loaded highest onto the first contrast from Model 2:  Items 2, 3, 4, 5 | 2 | LTCQROLE | 0.96 | 0.94 |
|  | 3 | LTCQPHYS | 0.94 | 0.91 |
|  | 4 | LTCQCONT | 1.11 | 1.10 |
|  | 5 | LTCQENJO | 0.95 | 1.00 |
| 5. 12 items - Remaining scale after removing 6 items as a separate scale and items 16 and 17 | 6 | LTCQHOME | 1.20 | 1.05 |
|  | 7 | LTCQSAF1 | 0.92 | 0.69 |
|  | 8 | LTCQSAF2 | 0.92 | 0.83 |
|  | 9 | LTCQSYMP | 0.97 | 1.11 |
|  | 10 | LTCQDEPE | 1.14 | 1.12 |
|  | 11 | LTCQLONE | 1.04 | 0.97 |
|  | 12 | LTCQSTIG | 0.96 | 0.90 |
|  | 13 | LTCQSERV | 1.27 | 1.09 |
|  | 14 | LTCQTREA | 1.25 | 1.36 |
|  | 15 | LTCQUNHA | 0.81 | 0.82 |
|  | 18 | LTCQSUPP | 1.09 | 1.18 |
|  | 19 | LTCQCONF | 0.85 | 0.76 |
| 6. 13 items – Reintroduced item 4 to remaining scale | 4 | LTCQCONT | 0.79 | 0.76 |
|  | 6 | LTCQHOME | 1.21 | 1.04 |
|  | 7 | LTCQSAF1 | 0.94 | 0.69 |
|  | 8 | LTCQSAF2 | 0.92 | 0.82 |
|  | 9 | LTCQSYMP | 1.02 | 1.18 |
|  | 10 | LTCQDEPE | 1.16 | 1.12 |
|  | 11 | LTCQLONE | 1.08 | 1.02 |
|  | 12 | LTCQSTIG | 1.01 | 0.95 |
|  | 13 | LTCQSERV | 1.30 | 1.12 |
|  | 14 | LTCQTREA | 1.28 | 1.42 |
|  | 15 | LTCQUNHA | 0.82 | 0.84 |
|  | 18 | LTCQSUPP | 1.12 | 1.24 |
|  | 19 | LTCQCONF | 0.82 | 0.74 |
| 7. 12 items – Removal of item 14 | 4 | LTCQCONT | 0.80 | 0.73 |
|  | 6 | LTCQHOME | 1.24 | 1.07 |
|  | 7 | LTCQSAF1 | 0.94 | 0.68 |
|  | 8 | LTCQSAF2 | 0.92 | 0.83 |
|  | 9 | LTCQSYMP | 1.05 | 1.21 |
|  | 10 | LTCQDEPE | 1.17 | 1.14 |
|  | 11 | LTCQLONE | 1.09 | 1.02 |
|  | 12 | LTCQSTIG | 1.04 | 0.97 |
|  | 13 | LTCQSERV | 1.39 | 1.19 |
|  | 15 | LTCQUNHA | 0.85 | 0.86 |
|  | 18 | LTCQSUPP | 1.14 | 1.29 |
|  | 19 | LTCQCONF | 0.75 | 0.73 |
| 8. 11 items – Removal of item 13 | 4 | LTCQCONT | 0.82 | 0.75 |
|  | 6 | LTCQHOME | 1.25 | 1.07 |
|  | 7 | LTCQSAF1 | 0.95 | 0.69 |
|  | 8 | LTCQSAF2 | 0.94 | 0.85 |
|  | 9 | LTCQSYMP | 1.06 | 1.27 |
|  | 10 | LTCQDEPE | 1.21 | 1.17 |
|  | 11 | LTCQLONE | 1.11 | 1.04 |
|  | 12 | LTCQSTIG | 1.09 | 1.01 |
|  | 15 | LTCQUNHA | 0.85 | 0.86 |
|  | 18 | LTCQSUPP | 1.16 | 1.29 |
|  | 19 | LTCQCONF | 0.84 | 0.76 |
| 9. 10 items – Removal of item 18 | 4 | LTCQCONT | 0.81 | 0.74 |
|  | 6 | LTCQHOME | 1.30 | 1.13 |
|  | 7 | LTCQSAF1 | 0.98 | 0.71 |
|  | 8 | LTCQSAF2 | 0.83 | 0.75 |
|  | 9 | LTCQSYMP | 1.08 | 1.29 |
|  | 10 | LTCQDEPE | 1.15 | 1.12 |
|  | 11 | LTCQLONE | 1.12 | 1.04 |
|  | 12 | LTCQSTIG | 1.11 | 1.03 |
|  | 15 | LTCQUNHA | 0.85 | 0.86 |
|  | 19 | LTCQCONF | 0.89 | 0.86 |
| 10. 9 items – Removal of item 9 | 4 | LTCQCONT | 0.80 | 0.76 |
|  | 6 | LTCQHOME | 1.28 | 1.16 |
|  | 7 | LTCQSAF1 | 0.93 | 0.69 |
|  | 8 | LTCQSAF2 | 0.90 | 0.84 |
|  | 10 | LTCQDEPE | 1.22 | 1.25 |
|  | 11 | LTCQLONE | 1.15 | 1.10 |
|  | 12 | LTCQSTIG | 1.13 | 1.10 |
|  | 15 | LTCQUNHA | 0.91 | 0.97 |
|  | 19 | LTCQCONF | 0.88 | 0.88 |
| 11. 8 items – removal of item 6 | 4 | LTCQCONT | 0.83 | 0.80 |
|  | 7 | LTCQSAF1 | 1.06 | 0.82 |
|  | 8 | LTCQSAF2 | 0.93 | 0.86 |
|  | 10 | LTCQDEPE | 1.21 | 1.20 |
|  | 11 | LTCQLONE | 1.13 | 1.06 |
|  | 12 | LTCQSTIG | 1.13 | 1.10 |
|  | 15 | LTCQUNHA | 0.90 | 0.95 |
|  | 19 | LTCQCONF | 0.90 | 0.89 |
